# Chemical Constituents from *Clematis delavayi* var. *spinescens*

**DOI:** 10.3390/molecules14114433

**Published:** 2009-11-05

**Authors:** Yang Li, Si-Feng Wang, Yan-Li Zhao, Ke-Chun Liu, Xi-Min Wang, Yong-Ping Yang, Xiao-Li Li

**Affiliations:** 1 Laboratory of Ethnobotany, Kunming Institute of Botany, Chinese Academy of Sciences, Kunming 650204, China; E-Mails: liyang@mail.kib.ac.cn (Y.L.); zhaoyanli@mail.kib.ac.cn (Y.L.Z.); 2 Institute of Tibetan Plateau Research at Kunming, Kunming Institute of Botany, Chinese Academy of Sciences, Kunming 650204, Yunnan, China; E-Mail: yangyp@mail.kib.ac.cn (Y.P.Y.); 3 Biology Institute of Shandong Academy of Sciences, Jinan 250014, Shandong, China; E-Mails: peakwang10@gmail.com (S.F.W.); hliukechun@keylab.net (K.C.L.); wangximin727@sohu.com (X.M.W.)

**Keywords:** coumarin, *Clematis delavayi* var. *spinescens*, NMR, antiangiogenic effects

## Abstract

A new coumarin, 7-hydroxy-4,6-dimethoxy-5-methylcoumarin (**1**), was isolated from the aerial parts of *Clematis delavayi* var. *spinescens* together with 17 known compounds. Their structures were identified by extensive spectral analysis, especially 2D NMR techniques. Antiangiogenic effects of all compounds were evaluated using a zebrafish model.

## 1. Introduction

The genus *Clematis*, belonging to the family Ranunculaceae, is a large genus with about 300 species. The roots and rhizomes of *Clematis* are traditionally used as an analgesic, abirritative, antibacterial, antiphlogistic, anticancer and diuretic agent. Crude extracts from plants of this genus showed diuretic [1], antimicrobial [2], anti-inflammatory [3] biological activities. Some triterpenoid saponins and alkaloids isolated from this genus showed cytotoxic [4], antibacterial [5], and antifungal [6] activities.

**Figure 1:**
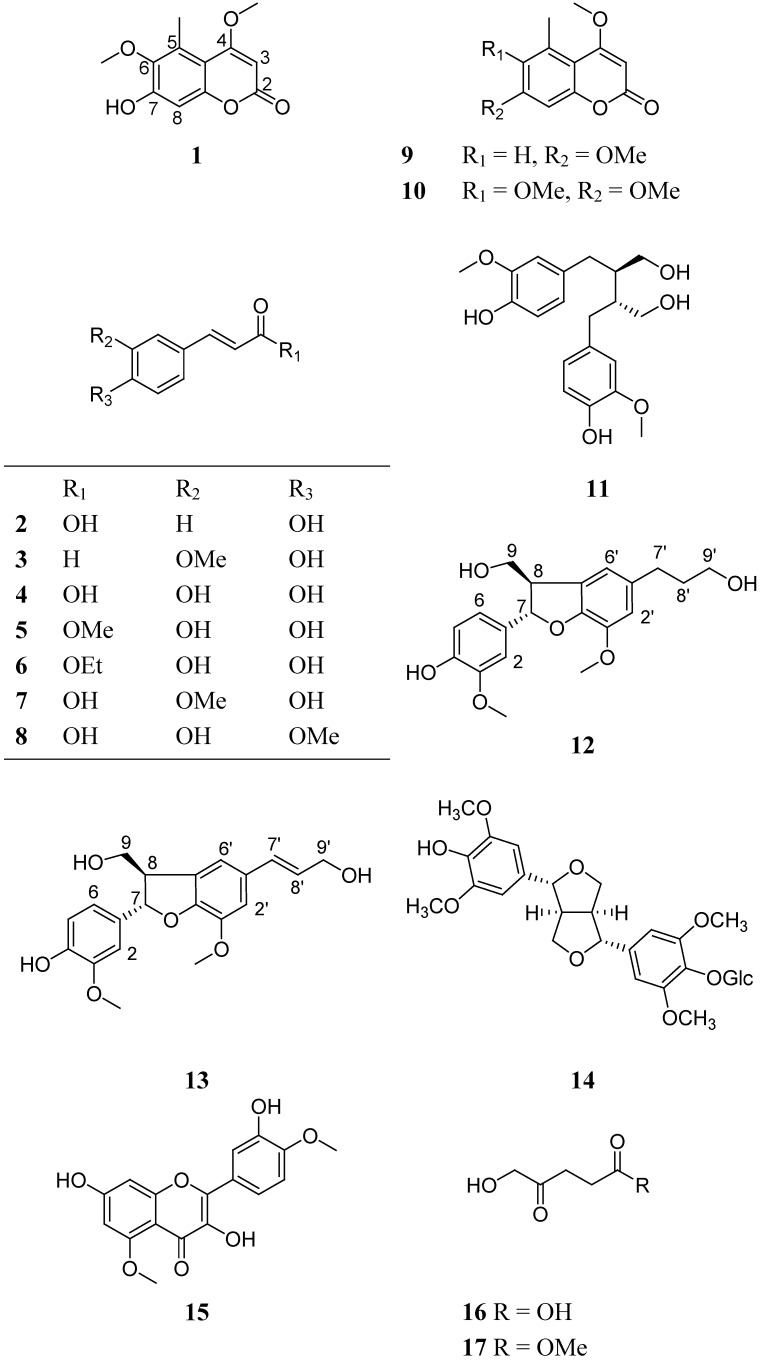
The structures of compounds **1-17.**

*Clematis delavayi* var. *spinescens* is an apically spinescent shrub, which is widely distributed in the dry valleys of the upper reaches of the Yangtze River in Southwest China [7]. Up to now, no phytochemical study on *C. delavayi* var. *spinescens* was reported. Our investigation on the aerial parts of this plant lead to the identification of a new coumarin, 7-hydroxy-4,6-dimethoxy-5-methylcoumarin (**1**), and 17 known compounds, including (*E*)-*para*-coumatic acid (**2**) [8], coniferaldehyde (**3**) [9], caffeic acid (**4**) [10], caffeic acid methyl eater (**5**) [11], ethyl caffeate (**6**) [12], ferulic acid (**7**) [17], isoferulic acid (**8**) [10], 4,7-dimethoxy-5-methylcoumarin (**9**) [14], 4,6,7-trimethoxy-5-methylcoumarin (**10**) [14], (–)-secoisolariciresinol (**11**) [15], (+)-dihydrodehydrodiconiferyl alcohol (**12**) [16], dehydrodiconiferyl alcohol (**13**) [17], (+)-syringaresinal-4′-*O*-β-D-glucopyranoside (**14**) [18], 3, 3′,7-trihydroxy-4′,5-di-methoxyflavone (**15**) [19], 5-hydroxy-4-oxopentanoic acid (**16**) [20], 4-carbonyl-5-hydroxy methyl valerate (**17**) [21] and daucosterol (**18**) (Figure 1). In addition, the antiangiogenic effects of all compounds were evaluated using a zebrafish model and none of them were bioactive in this assay.

## 2. Results and Discussion

Compound **1 **was isolated as white powder. Its molecular formula was established as C_12_H_12_O_5_ on the basis of HRESIMS ([M + H]^+^, found 237.0762, calcd 237.0762). The ^1^H- and ^13^C-NMR data (Table 1), coupled with UV absorptions at 208, 291, 325 nm and IR bands at 1,704 and 1,618 cm^-1^, revealed that **1** has a coumarin skeleton. 

**Table 1:** The NMR data of **1** (in DMSO-d_6_).^ a^

Position	*δ*_C_	*δ*_H_	Position	*δ*_C_	*δ*_H _
2	161.9 (s)	–	8	101.6 (d)	6.64 (s)
3	87.2 (d)	5.59 (s)	9	151.6 (s)	–
4	169.5 (s)	–	10	106.1 (s)	–
5	129.5 (s)	–	OMe-(4)	56.6 (q)	3.90 (3H, s)
6	143.5 (s)	–	OMe-(6)	60.0 (q)	3.62 (3H, s)
7	154.4 (s)	–	Me-(5)	14.0 (q)	2.50 (3H, s)

^a 1^H- and ^13^C-NMR spectra were obtained at 500 and 125 MHz, respectively.

**Figure 2:**
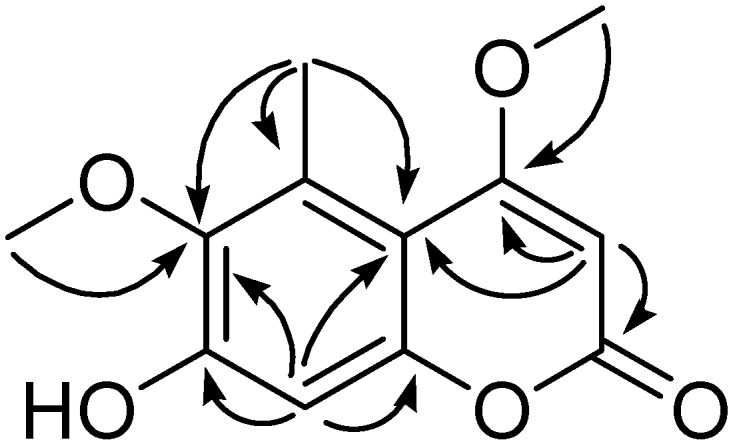
Key HMBC (H → C) correlations of **1**.

Only two downfield signals (*δ*_H_ 6.64 s, 5.59 s) were observed in the ^1^H-NMR spectrum of **1**, suggesting that **1** was a tetrasubstituted coumarin. The ^13^C-NMR spectrum showed the presence of one carbonyl carbon, two methines, six quaternary carbons, one methyl group, and two methoxyl groups. Initial comparison of ^1^H-NMR and ^13^C-NMR data of **1** with those of known compound 4,6,7-trimethoxy-5-methylcoumarin (**10**) [8] showed compound **1** is very similar to **10**. The big difference is the presence of only two methoxyl groups in **1**, which is one less than those of **10**. The methyl group was assigned to be located at C-5 by the HMBC correlations of proton of the methyl group (*δ*_H _2.48, s, 3H) to C-5 (*δ*_C_ 129.5), C-6 (*δ*_C_ 143.5) and C-10 (*δ*_C _106.1) (Figure 2). 

A methoxyl group was established to be located at C-4 by the HMBC correlation of OMe (*δ*_H _3.90, s, 3H) to C-4 (*δ*_C _169.5), which was also supported by the ROESY correlation between the proton signal of this methoxyl group and H-3. The HMBC correlation of OMe (*δ*_H _3.62, s, 3H) to C-6 (*δ*_C _143.5) showed that another methoxyl group was located at C-6. According to its molecular formula, the remaining substitute group was assigned to be a hydroxyl group, which was determined to be located at C-7 by the downfield chemical shift of C-7 (*δ*_C _154.4), and the side-by-side NMR data comparison of **1** with **10**. Therefore, the structure of **1** was elucidated as 7-hydroxy-4, 6-dimethoxy-5-methylcoumarin.

The antiangiogenic activities of all compounds were evaluated using a zebrafish model, in terms of the inhibition on the growth of intersegmental vessels, with PTK787 as a positive control (IC_50_ 0.15 μg/mL) [22]. All compounds in the assay showed bioactivity with IC_50_ values above 5 µg/mL.

## 3. Conclusions

A new coumarin, 7-hydroxy-4, 6-dimethoxy-5-methylcoumarin (**1**) and 17 known compounds were isolated from the EtOAc extract of *C. delavayi* var. *spinescens* aerial parts. Their structures were identified by extensive spectral analysis, especially 2D NMR techniques. Most of these compounds are belonging are phenolics, including coumarins, lignans and flavonoids. The antiangiogenic activities of all compounds were evaluated using a zebrafish model, and did not show obvious bioactivity, with IC_50_ values more than 5 µg/mL.

## 4. Experimental Section

### 4.1. General

1D and 2D NMR spectra were recorded on Brucker AM-400 and DRX-500 spectrometers. Unless otherwise specified, chemical shift (*δ*) were expressed in ppm with reference to the solvent signals. MS were performed on a VG Autospec-3000 spectrometer under 70 eV. Optical rotation was measured with a Horiba SEPA-300 polarimeter. A Bio-Rad FTS-135 spectrophotometer was used for scanning IR spectroscopy of compounds with KBr pellets. Column chromatography was performed on silica gel (200–300 mesh, Qingdao Marine Chemical Inc., Qing-dao, Peoples Republic of China) and silica gel H (10–40 μm, Qingdao Marine Chemical Inc.). Fractions were monitored by TLC and spots were visualized by heating plates spraying with 10% H_2_SO_4_ in EtOH.

### 4.2. Plant Materials

The aerial parts of *C. delavayi* var. *spinescens* were collected from Derong County of Sichuan Province. The plant material was identified by Prof. Yongping Yang. A voucher specimen (No. LY 200709002) was deposited at Kunming Institute of Botany, Chinese Academy of Sciences.

### 4.3. Extraction and Isolation

The air-dried aerial parts of *C.* *delavayi* var. *spinescens* (6.0 kg) were powdered and extracted with 70% aqueous acetone (3 × 15 L) for 24 h at room temperature and concentrated *in vacuo* to give a crude extract (100 g), which was suspended in H_2_O and partitioned with EtOAc. The EtOAc extract was evaporated and the residue (90 g) was subjected to open column chromatography over MCI-gel CHP-20P eluting with 95% ethanol. The eluent from 95% ethanol (68 g) was concentrated *in vacuo* and subjected to column chromatography over silica gel (200–300 mesh) eluting with petroleum ether and acetone (1:0, 4:1, 2:1, 1:1, 1:2 and 0:1) to afford fractions A–E. Fraction B was subjected to Sephadex LH-20 (CHCl_3_/MeOH 1:1) and column chromatography over silica gel (petroleum ether/acetone) to yield **10** (10.2 mg), **16** (135.0 mg). Fraction C was chromatographed on Sephadex LH-20 (CHCl_3_/MeOH 1:1) and silica gel (CHCl_3_/MeOH) columns to yield **1** (46.1 mg), **2** (9.0 mg), **5** (1.0 mg), **7** (16.2 mg), **8** (6.3 mg), **11** (5.1 mg), **12** (2.0 mg), **13** (2.1 mg). Fraction D was also subjected to sephadex LH-20 (CHCl_3_/MeOH 1:1) and column chromatography over silica gel (CHCl_3_/MeOH) to yield **3** (3.2 mg), **4** (374.0 mg), **6** (6.1 mg), **9** (3.1 mg), **14** (25.2mg), **15** (1.0 mg), **17** (2.3 mg), **18** (47.5 mg).

*Compound* **1**: white powder; UV_ max _(MeOH): 208, 291, 325 nm; IR (KBr); *υ*_max_ 3424, 1704, 1691, 1618, 1563 cm^−1^; For ^1^H- and ^13^C-NMR see Table 1. HRESIMS ([M + H]^+^, found 237.0762, calcd 237.0762).

### 4.4. Antiangiogenesis Assay *[22]*

Stock solutions (10 mg/mL) of all samples were prepared by dissolving the test compounds in 100% DMSO. These solutions were diluted in sterile salt water (5 mM NaCl, 0.17 mM KCl, 0.4 mM CaCl_2_, 0.16 mM MgSO_4_) to obtain solutions with the test compounds dissolved in 0.1% DMSO. These solutions were aliquoted into 96-well plates, and embryos at 24 hpf (hours post-fertilization) were also transferred randomly into the above wells. After 24-h treatment, the intersegmental vessels of embryos were visualized with methods of green fluorescent protein labeling and endogenous alkaline phosphatase staining. The antiangiogenic activities of compounds were calculated from the inhibition ratio of angiogenesis. PTK787 was used as the positive control.
